# A Retrospective Cohort Study of the Potency of lipid-lowering therapy and Race-gender Differences in LDL cholesterol control

**DOI:** 10.1186/1471-2261-11-58

**Published:** 2011-09-30

**Authors:** Barbara J Turner, Christopher S Hollenbeak, Mark Weiner, Simon SK Tang

**Affiliations:** 1 Division of General Internal Medicine, University of Pennsylvania, 423 Guardian Drive, Philadelphia, PA, 19104, USA; 2 Departments of Surgery and Public Health Sciences, Penn State College of Medicine, 600 Centerview Drive, A210, Hershey, PA, 17033, USA; 3 Pfizer, Inc., 235 East 42nd Street, New York, NY, 10017, USA

**Keywords:** dyslipidemia, anticholesterolemic agents, healthcare disparities, survival analysis

## Abstract

**Background:**

Reasons for race and gender differences in controlling elevated low density lipoprotein (LDL) cholesterol may be related to variations in prescribed lipid-lowering therapy. We examined the effect of lipid-lowering drug treatment and potency on time until LDL control for black and white women and men with a baseline elevated LDL.

**Methods:**

We studied 3,484 older hypertensive patients with dyslipidemia in 6 primary care practices over a 4-year timeframe. Potency of lipid-lowering drugs calculated for each treated day and summed to assess total potency for at least 6 and up to 24 months. Cox models of time to LDL control within two years and logistic regression models of control within 6 months by race-gender adjust for: demographics, clinical, health care delivery, primary/specialty care, LDL measurement, and drug potency.

**Results:**

Time to LDL control decreased as lipid-lowering drug potency increased (P < 0.001). Black women (N = 1,440) received the highest potency therapy (P < 0.001) yet were less likely to achieve LDL control than white men (N = 717) (fully adjusted hazard ratio [HR] 0.66 [95% CI 0.56-0.78]). Black men (N = 666) and white women (N = 661) also had lower adjusted HRs of LDL control (0.82 [95% CI 0.69, 0.98] and 0.75 [95% CI 0.64-0.88], respectively) than white men. Logistic regression models of LDL control by 6 months and other sensitivity models affirmed these results.

**Conclusions:**

Black women and, to a lesser extent, black men and white women were less likely to achieve LDL control than white men after accounting for lipid-lowering drug potency as well as diverse patient and provider factors. Future work should focus on the contributions of medication adherence and response to treatment to these clinically important differences.

## Background

Reducing low density lipoprotein (LDL) cholesterol to levels set by National Cholesterol Education Program Adult Treatment Panel (ATP) III guidelines decreases the risk of death from cardiovascular disease [1] and is cost-effective [2]. Analyses of National Health and Nutrition Examination Surveys (NHANES) from 1999 to 2006 reveal persistent gender and racial differences in meeting these standards [3]. Several studies have implicated physician's treatment of dyslipidemia as contributing to these differences in LDL control. Among diabetic patients, black patients were less likely to be prescribed statin therapy than white patients and white men were more likely to achieve LDL control than either women or black men [4]. An analysis of increases in doses of lipid-lowering drugs to achieve LDL control reported no difference by race but women had fewer dose increases than men [5]

Most studies of racial differences in achieving LDL control have been cross-sectional and do not consider the potency of prescribed lipid-lowering drugs [6-8]. To evaluate the impact of type and total dose of lipid lowering therapy on racial and gender disparities in LDL control, we adapted a methodology developed previously to assess the potency of antihypertensive drug therapy [9]. Among primary care patients at increased risk of cardiovascular disease because of hypertension and elevated LDL cholesterol, we hypothesized that race and gender differences in achieving LDL control would be reduced after accounting for lipid-lowering drug potency and baseline LDL values.

## Methods

### Study Sample

Study patients received longitudinal care over a four-year period (1/1/03 through 1/1/07) in 6 primary care practices (one family medicine and 5 general internal medicine) affiliated with an academic medical center in Philadelphia, PA. All practices used EPIC electronic medical record system that offers: demographics, physiologic measures, clinical diagnoses, tobacco use, laboratory data, visit attendance data, prescribed medications, and insurance information. Electronic prescribing is required and free samples prohibited. We linked data on providers' gender, race, and training level (i.e., resident, attending, and nurse practitioner) from certification and departmental sources.

**Figure 1:**
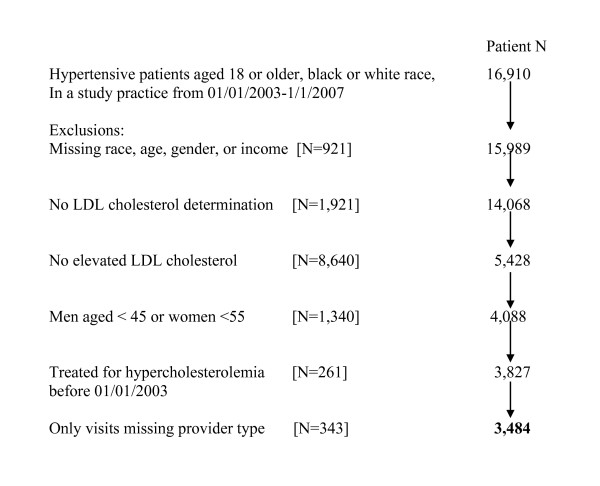
**Derivation of Study Cohort of Older Hypertensive Patients with Dyslipidemia**.

For research on hypertension [10] and cholesterol [11] quality of care, we developed a cohort of hypertensive black or white patients aged ≥ 18 [N = 16,910] who have been treated for at least 6 months in a study practice. In this study, we included only older persons whose ATP III LDL goal is < 130 mg/dl or < 100 for those with cardiovascular disease, diabetes, or a diabetes equivalent condition [12] (Figure 1). All study subjects were followed in study practices at least 6 months after their first high LDL.

### Outcome Variables

The primary study outcome was the time in days from the initial elevated LDL value after 1/03 until LDL control per ATP III guidelines (i.e., < 130 mg/dl if moderate risk or < 100 mg/dl if high risk [1]) was achieved. We also examined the proportion of patients who achieved control within 6 months after the high baseline LDL.

### Predictor Variables

Our primary predictor was a four level race-gender variable (i.e., women and men grouped as white or black). Demographics included age and median household annual income based on residence zip code in 2000 United States census data (http://www.census.gov). We extracted all prescriptions (i.e., statins, fibrates, nicotinic acid derivatives, bile acid sequestrants, and cholesterol absorption inhibitors) during the study timeframe. To calculate the daily potency of lipid-lowering therapy, we assigned a weight to each lipid-lowering drug and dose based on published data and reports on reductions in LDL cholesterol (Table 1) [13-15]. A weight of 1.00 was assigned to atorvastatin 10 mg per day (the most frequently prescribed dose) that reduces LDL cholesterol by a mean of 34-38%. We then determined the relative weights for all prescribed lipid-lowering drugs and doses based on the mean reduction in LDL. For example, assuming a 36% LDL reduction for atorvastatin 10 mg (the midpoint for 34-38%), pravastatin 10 mg, which has an expected LDL reduction of 21.5%, received a weight of .215/.36 = 0.597. Daily potency weights for drug combinations such as atorvastatin plus ezetimibe were summed for each day.

**Table 1:** Relative Daily Potency of the HMG-CoA Reductase Inhibitors [Statins] and Non-statin Lipid-lowering Drugs

Drug	Daily dose range in study population	Effect on LDL Cholesterol* [% decrease]	Relative potency† of lowest and highest dose
Statins			
Atorvastatin	5-80 mg	32-50%	0.89, 1.39
Fluvastatin	20-80 mg	22-35%	0.61, 0.97
Lovastatin	10-120 mg	21-50%	0.58, 1.39
Pravastatin	5-320 mg	15-40%	0.42, 1.11
Rosuvastatin	2.2-60 mg	39-60%	1.08, 1.67
Simvastatin	5-160 mg	22-50%	0.61, 1.39
Non-Statins			
Cholestyramine	4-16 g	4-9%	0.10, 0.25
Colesevelam	625-3750 mg	5-10%	0.14, 0.28
Colestipol	1-10 g	5-10%	0.14, 0.28
Ezetimibe	5-30 mg	10-20%	0.28, 0.56
Fenofibrate	27-320 mg	5-10%	0.14, 0.28
Gemfibrazole	300-1200 mg	5-10%	0.14, 0.28
Niacin	250-3000 mg	4-9%	0.10, 0.25
Omega	3-12 mg	4-5%	0.10, 0.14

* References 13-15

† Atorvastatin 10 mg per day assigned a daily potency weight of 1.

To calculate total lipid-lowering drug potency and allow for the dynamic nature of our follow-up, we adapted a method developed by Bailey and colleagues [9] to assess antihypertensive drug potency and previously used in our research [16]. First, the duration of each prescription was calculated from the number of prescribed lipid-lowering medication pills and refills and, in case of overlapping prescriptions, we counted the most recent. To calculate total potency, we multiplied the potency weight of each day's lipid-lowering therapy by days of prescribed drug(s). For example, a patient taking atorvastatin 10 mg (daily potency weight = 1) for 60 days was assigned a value of 60 for total potency but the same value would be assigned to a 10 mg dose of pravastatin (daily potency weight = 0.597) prescribed for 101 days. Persons who received no treatment at all had a total potency of 0. We created an indicator for the presence of lipid-lowering therapy at the start of the study timeframe. For analyses of prescribed lipid-lowering therapy, we examined categorical and continuous specifications of drug potency.

We categorized baseline elevated LDL cholesterol by quartile. We also created indicators for the number of LDL determinations in the first 6 months after the baseline elevated LDL. Other clinical measures include: diabetes (i.e. ICD-9-CM 250.xx at two visits or hemoglobin A1c ≥ 7 mg/dl); renal insufficiency (i.e., creatinine > 2); other vascular diseases (i.e. coronary artery disease, peripheral vascular disease, cerebrovascular disease); and 28 non-cardiovascular comorbidities (e.g. arthritis, gastroesophageal reflux/gastritis) as reported previously [10]. Smoking status was categorized as currently smoking, not smoking, or not recorded. We calculated the maximum number of concurrent antihypertensive drugs prescribed within the study timeframe.

Health care variables included: insurance type and attendance to scheduled primary care visits within a 6-year interval (i.e., 1/02 to 1/07 categorized in quartiles). Primary care provider characteristics were: gender, race, type, and workload (in quartiles) based on the maximum annual patient visits in the study timeframe.

### Analyses

The cohort was followed for at least 6 months to a maximum of 24 months to determine if LDL cholesterol control per ATP III guidelines was achieved. We conducted two types of analyses. Our first outcome was time until LDL cholesterol control examined using survival analysis methods with Cox proportional hazards models, censoring if a subject did not achieve LDL control by 6 months after the last visit or the end of the study. We examined Kaplan-Meier curves of time until LDL control for all key variables as well as proportional hazards assumptions. For the second outcome of LDL control by 6 months after the initial elevated LDL, we estimated logistic regression models.

Among treated persons, lipid-lowering drug potency was examined both as a continuous variable and in two categories: low (a mean daily potency weight of 1 for less than half of the mean number of treated days [n = 450]) versus high (a mean potency weight of 1 for more than half of the mean treated days). Persons with no treatment were considered as a third category.

In a series of Cox proportional hazards models, we sequentially added 5 sets of variables to assess changes in the hazard ratios for each race-gender group versus white men. The sets of variables were: demographics; clinical; health care; primary care provider and specialty care; baseline LDL, frequency of LDL checks, and lipid-lowering drug potency. We included an indicator of time [days] since the initial LDL was obtained. We also fit parametric Weibull survival models with physician level frailties to examine the effect of clustering on primary care provider but the conclusions were similar to reported models and are not shown.

The logistic regression models adjust for all study variables. Sensitivity analyses examined stability of the gender-race association in logistic models for subsets of subjects with the same insurance (Medicare or commercial), neighborhood income level, or age group. We also estimated models among only subjects with diabetes or another condition warranting a lower [< 100 mg/dl] LDL goal.

### Role of the Funding Source

Research funding was provided by Pfizer, Inc. to the University of Pennsylvania. Authors from the University of Pennsylvania and Penn State College of Medicine conducted all analyses and wrote the manuscript. One author is a Pfizer employee, but he did not have direct access to study data. This author obtained study funding, contributed to the study design and reviewed the manuscript. Pfizer had no role in the decision to submit the manuscript for publication. This project was approved by the University of Pennsylvania Institutional Review Board.

## Results

The derivation of the study cohort is shown in Figure 1. Eighty-nine percent of patients aged 18 or older had baseline LDL cholesterol determination and, of these, 39% had an elevated level. Among the 3,484 study subjects with elevated LDL cholesterol, women were older than men and black subjects had lower income levels than white subjects. Women were more likely to be Medicare-enrolled than men and black patients were more likely to be Medicaid-enrolled than white patients (Table 2).

**Table 2:** Characteristics of Four Race-Gender Groups of Primary Care Hypertensive Patients with High Baseline LDL Cholesterol

Characteristic	Black women (N = 1,440)	Black men (N = 666)	White women (N = 661)	White men (N = 717)	P Value
Baseline LDL cholesterol [mg/dl], mean [SD]	142.8 (29.9)	138.9 (28.0)	144.0 (24.3)	137.2 (23.4)	< 0.001
LDL control by 6 months after baseline, %	16.8	18.9	20.3	28.5	< 0.001

LDL cholesterol tests within 6 months after baseline, N [SD]	0.85 (0.75)	0.90 (0.79)	0.80 (0.77)	0.92 (0.90)	0.026

**Sociodemographic**					

Age [years], mean [SD]	68.4 (9.7)	61.4 (11.1)	68.4 (9.9)	62.3 (10.5)	< 0.001
Median income in zipcode of residence, mean $ [SD]	28,360 (10,904)	30,692 (12,825)	57,004 (23,790)	58,680 (23,464)	< 0.001
Insurance type [%]					
Commercial	46.4	60.7	57.5	72.5	< 0.001
Medicaid	17.4	14.9	2.0	2.2	
Medicare	35.4	22.8	40.2	24.8	
Self pay	0.8	1.7	0.3	0.4	

**LDL Cholesterol**					

Baseline LDL [mg/dl], mean [SD]	142.8 (29.9)	138.9 (28.0)	144.0 (24.3)	137.2 (23.4)	< 0.001
LDL tests within 6 months mean [SD]	0.85 (0.75)	0.90 (0.79)	0.80 (0.77)	0.92 (0.90)	0.026
LDL control by 6 months after baseline, %	16.8	18.9	20.3	28.5	< 0.001
**Clinical**					

Vascular disease, %	30.1	28.2	21.2	21.1	< 0.001
Diabetes, %	41.5	46.2	19.5	31.1	< 0.001
Renal insufficiency, %	13.3	21.9	3.3	9.6	< 0.001
Unrelated comorbidities, #	6.3	5.4	5.5	4.9	< 0.001
Tobacco use, %					
Current	11.2	14.9	5.0	10.3	< 0.001
No	55.3	45.2	70.8	54.7	
Not recorded	33.5	39.9	24.2	35.0	

**Lipid-lowering Drug Therapy**					

Treated (%)	57.4%	54.7%	52.5%	53.7%	0.134
Potency per day of treatment, mean (SD)	0.96 (0.26)	0.97 (0.25)	0.90 (0.25)	0.91 (0.3)	< 0.001
Duration of treatment, days, mean (SD)	964.5 (550.3)	919.6 (537.6)	887.3 (544.9)	911.7 (548.4)	0.01
Total potency, categorical (treated only)					
None, %	42.6	45.4	47.5	46.3	
Lower [≤840],%	26.5	28.5	26.5	30.3	0.007
Higher [> 840],%	31.0	26.1	26.0	23.4	

**Health Care**					

Primary care arrived visits per year, mean (SD)	6.3 (4.2)	6.5 (4.6)	5.8 (3.7)	5.7 (6.1)	0.002
Kept < 60% of scheduled visits, %	19.7	20.7	13.0	10.2	< 0.001
Maximum concurrent anti-hypertensive drugs, mean N (SD)	2.5 (1.3)	2.3 (1.3)	1.9 (1.1)	1.8 (1.2)	< 0.001

**Provider (N)**					

Race, %					
White (157)	60.7	62.5	90.6	91.2	< 0.001
Asian (27)	11.7	7.7	3.3	2.5	
Other Minority (18)	27.6	30.0	6.1	6.3	
Gender, %					
Female (110)	60.3	47.3	53.7	31.3	< 0.001
Type, %					
Attending (74)	56.7	61.0	69.6	73.1	< 0.001
Resident (105)	17.0	16.2	7.9	8.4	
Nurse practitioner/Physician assistant (23)	26.3	22.8	22.5	18.6	
Annual arrived visits to	1916.8	1980.1	2009.0	2202.7	< 0.001
provider, mean N (SD)	(1464.7)	(1454.6)	(1207.5)	(1404.4)	

Women had higher baseline LDL levels than men but, within each gender group, the mean LDL values did not differ by race (Table 2). Time until a follow-up LDL determination did not significantly differ by race-gender group (P = 0.10) but men had significantly more LDL determinations within 6 months after the baseline elevated LDL cholesterol than women. LDL control at 6 months after baseline differed significantly by race-gender group but was poorest for black women.

Higher proportions of black patients were diagnosed with vascular disease, diabetes, or renal insufficiency than white patients. Black women had more non-cardiovascular comorbid conditions than the other race-gender groups. Current tobacco use was more prevalent in black men. Fifty-five percent of the cohort was prescribed lipid-lowering therapy during the study timeframe; black women were the most likely to be treated of the four race-gender groups. Among those who were treated, black women and men were prescribed more potent lipid-lowering medications than white women or men. Black women were also treated for more days than the other gender-race groups and had higher lipid-lowering drug potency.

In regard to health care, white patients had fewer annual arrived visits but black patients were less adherent to scheduled visits. Black patients were prescribed more antihypertensive medications. Most of the 202 primary care providers were white and more than half were women. Attending physicians delivered care to the majority of patients but treated fewer black patients.

**Figure 2:**
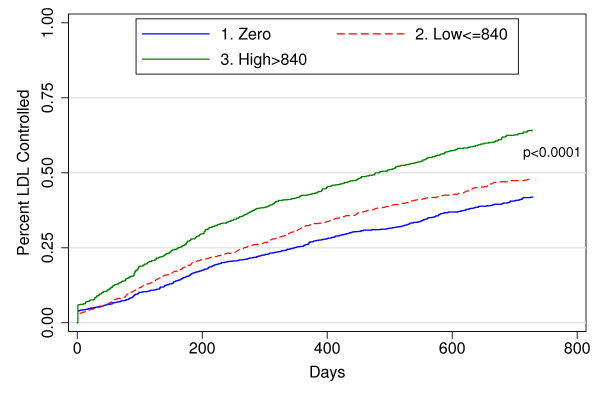
**Time Until LDL Cholesterol Control for Four Race-Gender Groups**.

Higher lipid-lowering drug potency was associated with shorter time to LDL control [P < 0.001] (Figure 2). Additional analyses showed that patients who were being prescribed lipid-lowering therapy at the time of the baseline elevated LDL did not differ in time to LDL control from those on no therapy at baseline (P = 0.83).

**Figure 3:**
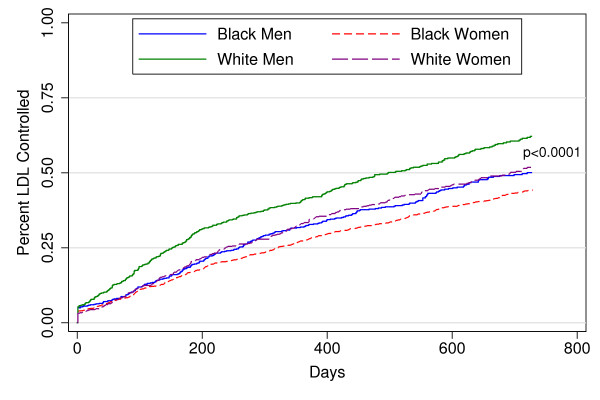
**Hazard Ratios for Time Until LDL Cholesterol Control for Four Race-Gender Groups by Categories of Total Potency of Lipid-lowering Drug Therapy**.* Categories: No Treatment; Low = 1-840 potency days; High = > 840 total lipid-lowering potency days [see methods], Adjusted for all the variables in Appendix Table 2.

**Figure 4:**
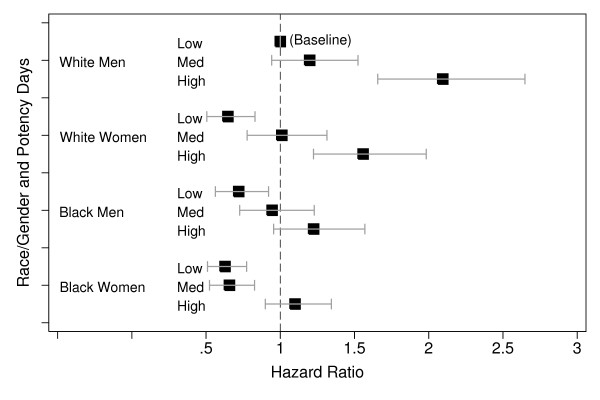
**Hazard Ratio for Achieving LDL Control Stratified by Race and Gender**.

**Figure 5:**
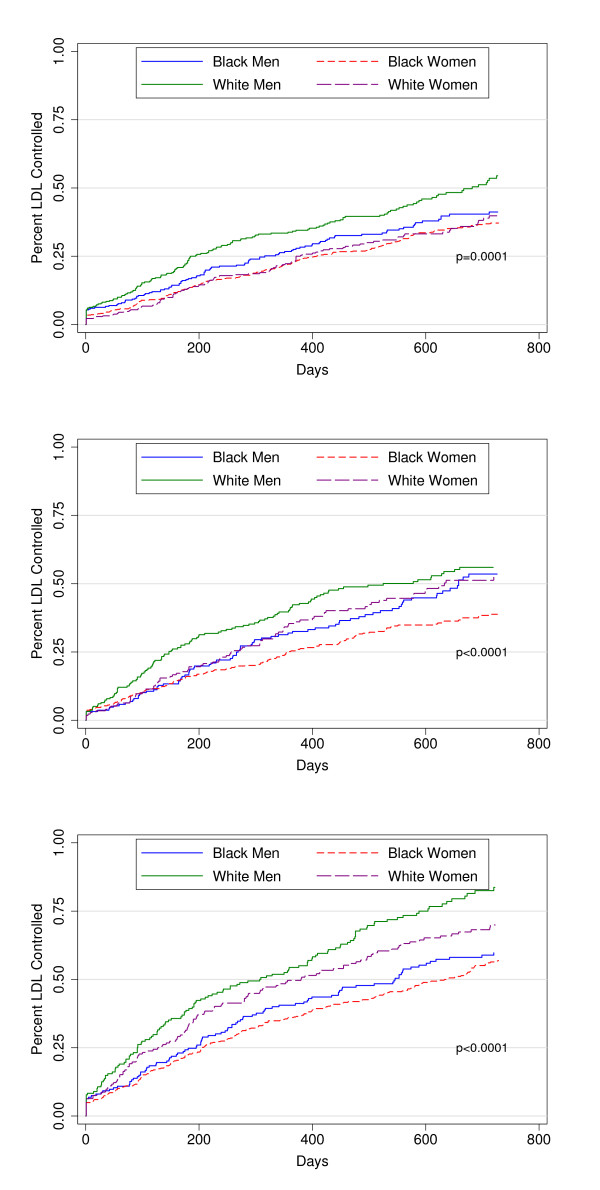
**Time to LDL Control Stratified by Race, Gender, and Potency of LDL Therapy**.

However, the mean unadjusted time until LDL control differed significantly (P < 0.001) by gender-race group: 540 days for black women, 505 days for white women, 510 days for black men, and 444 days for white men (Figure 3). Note that these times are shorter than the total time on therapy reported in Table 2 since patient remained on lipid lower therapy even after achieving LDL control. At each potency level, black women had a lower unadjusted hazard of achieving control than white men (Figure 4). Compared with white men, the unadjusted hazards of achieving LDL control were reduced by 39% for black women and by 25% for black men and 28% for white women (Table 3). Adjusting for demographic, clinical, health care and provider characteristics produced little change in these results. Adjusting for baseline LDL cholesterol, frequency of LDL checks (continuous), and lipid-lowering drug potency moderated these effects somewhat but the hazards of LDL control remained significantly lower for all three groups versus white men (Table 3). In the final model, high potency lipid-lowering therapy showed one of the strongest positive associations with LDL control of all covariates with an adjusted hazard of 1.85 (95% CI: 1.63, 2.1) for high potency relative no treatment (Table 4). Racial disparities in LDL control were most evident for subjects receiving higher potency therapy (Figure 5). In separate models among persons who only have diabetes or another risk factor with a lower LDL control standard (< 100 mg/dl), gender-race differences was similar to those in the entire cohort.

**Table 3:** Association of Race-Gender Groups with Time until LDL Cholesterol Control in Cox Proportional Hazards Models Adjusted for Sequentially Added Sets of Variables

	Variables Added to Model					
	**Unadjusted**	**Socio-** **demographic**	**Clinical** **Comorbidities**	**Health Care**	**Primary Care Provider**	**LDL Management**

	Hazard Ratio [95% CI]					

Race-gender Group						
Black Women	0.61 [0.53-0.69]‡	0.65 [0.56-0.76]‡	0.63 [0.54-0.74]‡	0.64 [0.54-0.75]‡	0.63 [0.53-0.74]‡	0.66 [0.56-0.78]‡
Black Men	0.75 [0.64-0.86]‡	0.78 [0.66-0.93]†	0.77 [0.65-0.91]†	0.79 [0.66-0.94]†	0.73 [0.63-0.86]†	0.82 [0.69-0.98]*
White Women	0.72 [0.62-0.83]‡	0.75 [0.64-0.87]‡	0.74 [0.64-0.86]‡	0.75 [0.64-0.87]‡	0.78 [0.65-0.93]‡	0.75 [0.64-0.88]†
White Men	1.0	1.0	1.0	1.0	1.0	1.0

*P < 0.05

† P ≤ 0.01

‡ P ≤ 0.001

Demographics [i.e., age, neighborhood income]; clinical comorbidities [i.e., vascular disease, diabetes, renal insufficiency, unrelated comorbidities, smoking]; health care [i.e., insurance, number of annual visits; visit adherence; antihypertensive medications]; primary care provider [i.e. type, race, gender, workload]; LDL management [i.e., baseline LDL, LDL checks within 6 months, and lipid-lowering drug potency].

**Table 4:** Final Model of Time until LDL Cholesterol Control

	Hazard	95% CI		
			
Characteristic	Ratio	*Lower*	*Upper*	P value
Gender-race				
Black women	0.66	0.56	0.78	< 0.0001
Black men	0.82	0.69	0.98	0.029
White women	0.75	0.64	0.88	< 0.0001
Baseline LDL cholesterol (quartile)				
Second	0.89	0.78	1.01	0.082
Third	0.67	0.58	0.77	< 0.0001
Fourth	0.48	0.42	0.56	< 0.0001
Total lipid-lowering drug potency				
Low	1.23	1.08	1.41	0.001
High	1.85	1.63	2.1	< 0.0001
LDL checks in first 6 months				
1-2	2.79	2.33	3.35	< 0.0001
3+	4.41	3.58	5.44	< 0.0001
Age [years]				
61-70	1.1	0.98	1.25	0.114
71-80	1.28	1.11	1.47	0.001
> 80	1	0.84	1.21	0.96
Median neighborhood (per $1000 increase)	1	1	1.01	0.016
Smoking status				
Current	0.99	0.84	1.18	0.934
Not reported	1.04	0.93	1.17	0.512
Vascular disease	0.8	0.7	0.92	0.001
Unrelated comorbidities (per comorbidity)	1.04	1.02	1.06	< 0.0001
Diabetes	0.81	0.72	0.9	< 0.0001
Chronic renal insufficiency	1.26	1.05	1.52	0.012
Insurance type				
Medicaid	0.87	0.72	1.04	0.128
Medicare	0.97	0.86	1.1	0.623
Self pay	0.85	0.42	1.71	0.641
High patient adherence to visits	1.31	1.13	1.51	< 0.0001
Antihypertensive drugs (N)	0.98	0.93	1.02	0.299
Provider type				
Resident	1.15	0.96	1.38	0.117
Other clinician	1.01	0.86	1.19	0.897
Provider race				
Asian	1.07	0.85	1.33	0.57
Black or other	0.99	0.84	1.16	0.877
Female provider	1.03	0.9	1.18	0.645
Provider workload (quartile of annual visits)				
Second	1.02	0.88	1.19	0.788
Third	0.96	0.82	1.12	0.58
Fourth	0.94	0.78	1.14	0.535
Patient visits (quartile of annual visits)				
Second	1.01	0.88	1.16	0.896
Third	1.09	0.95	1.25	0.24
Fourth	1.01	0.87	1.18	0.867

Analysis of the adjusted odds of LDL control by 6 months showed similar associations with a fully adjusted odds ratios for black women of 0.53 (95% CI: 0.40 - 0.71), for white women of 0.64 (95% CI: 0.49 - 0.85), for black men of 0.65 (95% CI: 0.48 - 0.89) versus white men. Even though black patients were more likely to have diabetes and other conditions that require a lower LDL goal (< 100 mg/dl), analyses restricted to only patients with these conditions showed similar odds ratios for each of the gender-race groups. Additional stratified analyses showed similar gender-race effects among: persons with Medicare insurance or commercial insurance, among persons with low income, and among persons with the same age category.

## Discussion

The US National Ambulatory Medical Care Survey from 2005 revealed marked inequities in assessment and management of dyslipidemia by respondents' race-ethnicity [17]. Among nearly 3,500 older primary care patients with hypertension and dyslipidemia, we use a novel approach to assessing the potency of prescribed lipid lowering therapy as a potential explanatory factor contributing to these inequities. Contrary to other studies that reported less treatment for dyslipidemia for women [18,19] or fewer lipid-lowering dose changes among treated women [5], the women in our study cohort were more likely to be treated for hyperlipidemia and prescribed more intensive lipid lowering therapy than men the same racial group. Indeed, black study women were the most likely to be treated for dyslipidemia and, when treated, were prescribed more potent treatment than black men, white women and white men. Mark et al. reported that black patients were less likely to have their lipid-lowering drugs switched, augmented, or titrated but did not examine achievement of LDL control goals [20]. In contrast, we find that, even though black patients received more intensive treatment, racial differences in achieving LDL cholesterol targets persisted. After two years, two-thirds of white men achieved LDL control versus only 50% or less of black men and women as well as white women. These sustained differences in LDL control are clinically significant [2] and may play a role in observed disparities in clinical outcomes of cardiovascular disease [21].

Our study focuses on the type and strength of prescribed lipid-lowering therapy but we could not evaluate the impact of counseling about a low cholesterol diet or adherence to the medication. In regard to unmeasured patient-related factors, inherent genetic differences may attenuate response to lipid-lowering therapy. However, in more controlled circumstances such as clinical trials, women and black men have responded to lipid-lowering therapy similarly to white men [22-24] and women [25]. Differences in adherence to lipid-lowering therapy may explain some of these associations; Chan and colleagues reported that men were more adherent to statins than women as were persons who lived in predominantly non-black compared with primarily black neighborhoods [26]. Among Medicaid enrollees, Litaker reported that black patients were 25% less likely to persist in taking lipid-lowering drugs than white patients [27]. Statin nonadherence may reflect deficient office based monitoring [28]; in our analysis, we accounted for adherence to office visits and frequency of LDL determinations.

Research has also implicated negative patient attitudes about lipid-lowering therapy [29,30] and limited understanding of the need for long-term therapy [29,31]. Increased patient education by the physician or an allied health personnel has been endorsed to address these attitudinal and knowledge barriers [32]. Our analyses did not adjust for restrictive pharmacy benefit plans or cost barriers to achieving LDL control [33]. Fortunately, generic statins have greatly reduced the cost of therapy [34] as have value-based drug benefit programs [35]. In sensitivity analyses, gender-race differences persisted among patients with Medicare insurance or those with commercial insurance. Chan and colleagues reported that adjusting for patient and physician characteristics as well as out-of-pocket costs did not significantly explain the variation in adherence to statins [26].

Overall, our cohort was prescribed relatively low potency lipid-lowering therapy, the equivalent of approximately simvastatin 20 mg per day. In a Norwegian study, the mean dose of prescribed lipid-lowering drugs was 25 mg for simvastatin and 22 mg for atorvastatin but, there too, doses were low [36]. Based on a recent simulation study, more tailored statin treatment would save lives if physicians initiated treatment with simvastatin 40 mg for persons at intermediate cardiovascular risk dose and atorvastatin 40 mg for persons at high risk [37]. The reasons for the conservative dosing of lipid-lowering drugs for our cohort are unknown but may reflect concerns about side effects at higher doses.

Our study has other limitations. First, the gender-race groups differed in multiple characteristics but, the differences in goal attainment persisted even when restricting the study analyses to persons with diabetes or other risk factor that requires a lower LDL standard. Second, our measure of total potency is the same for persons who take high doses for a short time or low doses for a longer time. However, racial differences persisted among only patients prescribed higher potency treatment. Third, the total pill burden may have negatively affected acceptance or persistence in taking lipid-lowering therapy. We did consider the number of prescribed antihypertensive drugs as well as the number of comorbidities that require more medications. In other analyses, patients with a greater number of concurrent medications were *more *likely to be adherent to statin therapy [38]. Fourth, we also could not consider the effect of therapeutic substitutions that can affect adherence [39]. Fifth, specialists might have prescribed these drugs but they share the same electronic medical record and medication reconciliation is required at each primary care visit.

Our study does not support the theory that differences in receiving lipid-lowering therapy or, when treated, potency of the prescribed therapy can account for widely observed racial- and gender-based differences in LDL cholesterol control. These data should direct attention to evaluating and addressing barriers to lipid-lowering drug adherence especially among black patients regardless of gender and white women. Promising approaches have been reported. For example, a health professional counseling by telephone significantly increased persistence with lipid-lowering medication but still only half of the subjects achieved LDL control within a year [40]. Finally, our study also reveals that primary care physicians may be prescribing sub-optimal doses of statin therapy demonstrating important opportunities to improve quality of care.

## Conclusions

In this study of 3,484 older hypertensive patients with dyslipidemia in 6 primary care practices, we found that black women, in spite of receiving the highest potency therapy, were less likely to achieve LDL control than white men. To a lesser extent, black men and white women were also less likely to achieve LDL control than white men after accounting for lipid-lowering drug potency as well as diverse patient and provider factors. Future work should focus on the contributions of medication adherence and response to treatment to these clinically important differences.

## Competing interests

This study was funded by a research grant from Pfizer, Inc., to the University of Pennsylvania. BT, CH, and MW received salary support from this funding. At the time the study was conducted, ST was an employee of Pfizer, Inc., and owned Pfizer stocks and stock options. None of the authors have any non-financial competing interests.

## Authors' contributions

BT conceived of the study and participated in its design, coordination, conduct, analysis, and drafted the manuscript. MW participated in the design of the study, conduct, and the interpretation of results. CH participated in the design of the study and performed the statistical analysis. ST participated in the conception and design of the study and secured funding. All authors revised the manuscript for content and read and approved the final manuscript.

## Pre-publication history

The pre-publication history for this paper can be accessed here:

http://www.biomedcentral.com/1471-2261/11/58/prepub
